# Morphological Changes to Fruit Development Induced by GA_3_ Application in Sweet Cherry (*Prunus avium* L.)

**DOI:** 10.3390/plants13152052

**Published:** 2024-07-25

**Authors:** Edoardo Vignati, Mario Caccamo, Jim M. Dunwell, Andrew J. Simkin

**Affiliations:** 1 Genetics, Genomics and Breeding, NIAB East Malling, New Road, Kent ME19 6BJ, UK; vignati.edoardo@gmail.com; 2 School of Agriculture, Policy and Development, University of Reading, Whiteknights, Reading RG6 6EU, UK; j.m.dunwell@reading.ac.uk; 3 Crop Bioinformatics, NIAB, 93 Lawrence Weaver Road, Cambridge CB3 0LE, UK; mario.caccamo@niab.com; 4 School of Biosciences, University of Kent, Canterbury CT2 7NJ, UK; 5 School of Life Sciences, University of Essex, Wivenhoe Park, Colchester CO4 3SQ, UK

**Keywords:** fruit development, cherry, gibberellin, parthenocarpy

## Abstract

Cherry (*Prunus avium*) fruits are important sources of vitamins, minerals, and nutrients in the human diet; however, they contain a large stone, making them inconvenient to eat ‘on the move’ and process. The exogenous application of gibberellic acid (GA_3_) can induce parthenocarpy in a variety of fruits during development. Here, we showed that the application of GA_3_ to sweet cherry unpollinated pistils acted as a trigger for fruit set and permitted the normal formation of fruit up to a period of twenty-eight days, indicating that gibberellins are involved in the activation of the cell cycle in the ovary wall cells, leading to fruit initiation. However, after this period, fruit development ceased and developing fruit began to be excised from the branch by 35 days post treatment. This work also showed that additional signals are required for the continued development of fully mature parthenocarpic fruit in sweet cherry.

## 1. Introduction

Sweet cherry trees (*Prunus avium*) are thought to be native to south Asia, Europe, and the isolated region of the western Himalayas [1,2], although they have become naturalised in North America and Australia, where they are widely cultivated due to their commercial importance [3,4]. Sweet cherry is a deciduous tree belonging to the Rosaceae family (https://sweetbiomics.com/) [5], and grows to between 15 and 32 m in height, producing an attractive fruit, classified as a drupe, that is 1–2 cm in diameter. In a commercial setting, cherry trees are grafted to commercial rootstocks, providing advantages in different regions (i.e., adapted for soil moisture content, soil types, and soil temperatures), often resulting in trees of a shorter stature. The rootstock Gisela 5 has established itself as the rootstock of choice, producing a tree growing to just 3 m in height with more manageable proportions (see Vignati et al. [3] for review).

Some of the most desired consumer attributes include firmness, sweetness, colour, and flavour intensity [6,7,8,9]; however, these consumer preferences often vary in different regions where different requirements determine what is considered to be a good cherry [10,11,12,13]. Cherry fruit enclose a large stone/seed, making fruit processing laborious and consumption by the consumer inconvenient, challenging to eat ‘on the move’, and potentially a dangerous choking hazard [3,14]. The availability of fruit lacking the stone/seed would be transformative for cherry producers, since stoneless/seedless fruits would be easier to process and increase consumer demand due to potential reductions in production costs [14]. 

Parthenocarpy is the induction of fruit development without pollination and ovule fertilisation, and largely depends on the coordinated action of hormones produced in unpollinated ovaries. It can be induced naturally or artificially [15]. Natural parthenocarpy has been reported in ninety-six Angiosperm taxa [15,16], and about half of the parthenocarpic species are trees that develop a drupe-type fruit [14]. Parthenocarpy can also be induced by the application of exogenous plant hormones such as gibberellins (GAs) [17]. Hormones play an essential role during fruit developmental processes, and it has been established for a long time that the application of phytohormones to emasculated flowers results in the formation of parthenocarpic fruit [18]. 

GAs consist of a group of terpenoid molecules (GA_1_, GA_3_, GA_4_, and GA_7_ are the bioactive GAs) that control and regulate the development of vegetative and reproductive organs [19,20,21,22,23,24]. Three GAs (GA_3_, GA_4_, and/or GA_7_) play crucial roles during fruit development, being positive modulators of several fruit-related features [22,23,25]. However, not all active GAs can induce parthenocarpy in all crops. For example, the exogenous application of GA_3_ can induce parthenocarpic fruit development in custard apple [26], apple (“Honeycrisp” cultivar) [27], grape [28,29], tomato [30], and citrus [31]. However, GA_3_ does not induce parthenocarpy in cucumber, where the simultaneous application of GA_4_ and GA_7_ (GA_4+7_) was needed [32]. GA_4+7_ has also been shown to induce parthenocarpy in pear [33,34]. 

GAs generate interest because they have been shown to induce parthenocarpy in the *Prunus* genus [35], in particular in peach (*P. persica*), apricot (*P. armeniaca*), almond (*P. dulcis*) [35], and more recently, in Chinese cherry (*P. pseudocerasus*) [36]. In almond (*P. dulcis*), apricot (*P. armeniaca*), and peach (*P. persica*), parthenocarpic fruit developed with success rates of 11.8%, 15.4% (500 ppm GA treatment), and 73.4%, respectively (50 ppm GA treatment applied twice). Moreover, the parthenocarpic peaches reached maturity approximately 10 days before the seeded fruit [35]. However, Crane et al. [35] also showed that *gibberellin* (GA) treatment did not produce any parthenocarpic fruit in *P. avium* (sweet cherry). This failure to induce parthenocarpy in sweet cherry may have been due to differences in the concentration or type of GA, given that Wen et al. [36] showed that emasculated *P. pseudocerasus* (Chinese cherry) pistils treated with a 300 mg/L GA_3_ solution was enough to induce the development of parthenocarpic fruit. 

In this manuscript, we evaluated the impact of treating emasculated sweet cherry flowers with two concentrations of GA_3_ (300 or 500 mg/L) to determine their impacts on fruit development and seed formation.

## 2. Results

### 2.1. Exogenous Application of Gibberellins Induces Putative Fruit Development in Sweet Cherry

**Figure 1:**
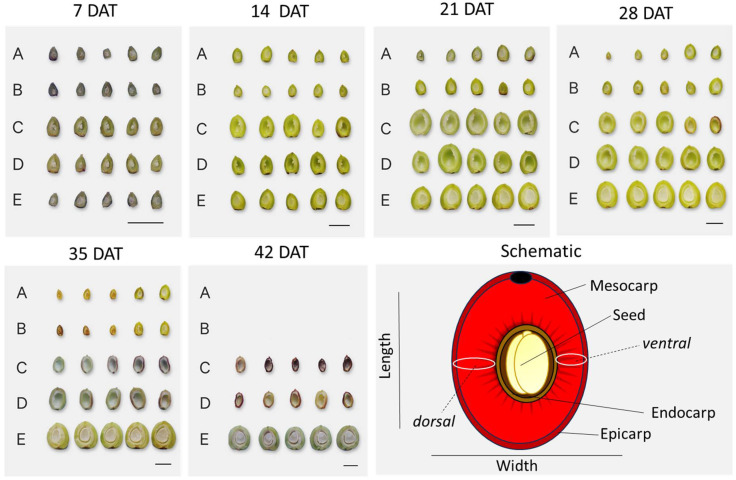
Panel that shows the (**A**) unpollinated ovaries (N.C.); (**B**) EtOH70%-treated ovaries; (**C**) 300 mg/L GA_3_-treated; (**D**) 500 mg/L GA_3_-treated; and (**E**) open pollinated (O.P.) Scale bars = 1 cm. DAT = days after treatment. Bottom right panel shows a schematic of measured features (adapted from Vignati et al. [14]. Fruit were divided down from the stalk to the tail. The thicker ovary/fruit wall side was designated “dorsal”, and the thinner opposite side as “ventral”. Five ovaries or fruits were photographed per treatment at every timepoint. Measurements of the dimensions were taken using ImageJ1 (https://imagej.net/).

Following exogenous treatment with GA_3_, unpollinated (negative control) and mock-treated (EtOH70%) pistils remained small and green without starting the fruit set process. From an external point of view, no visible difference between these two treatments was observed between 7 days after treatment (DAT) and 35 DAT (Figure 1A,B). The application of EtOH70% to the unpollinated pistils seems to have had no evident effect on their survival. By 42 DAT, no collection of unpollinated or mock-treated ovaries was possible, because all the ovaries wilted and abscised from the branches.

In both of the GA_3_ treatments, ovary enlargement of the samples was promoted by 7 DAT, similar to that observed in the open-pollinated controls. This enlargement was not caused by the presence of a developing seed, since the fruits of the GA_3_-treated samples were empty inside, whereas the seed was visible in the fruit of the open-pollinated samples (Figure 1; 7 DAT). In addition, at 7 DAT, slightly shrunk ovules were observed. After this time, the ovules either aborted, remained as traces, or disappeared completely. 

By 28 DAT, the GA_3_-treated putative developing fruits started to wilt by shrinking, and red and purple pigments started to accumulate, which became more evident by 35 DAT. This phenomenon was observed in both the 300 mg/L- and 500 mg/L-treated samples (Figure 1C,D). At 42 DAT, the observations were stopped, since the GA_3_ ovaries were shrivelled and had begun to be excised from the branch (Figure 1C,D). The open-pollinated ovaries started to increase in size by 7 DAT (Figure 1E), at a similar rate as the GA_3_-treated samples, but while the GA_3_-treated samples began to wilt and die by 35 DAT, the open-pollinated samples continued the fruit development process until maturity. 

In the open-pollinated (O.P) samples, the lumen of the fruit was filled with the developing seed, which appeared pulpy at every time point. The walls of both the GA_3_-treated putative developing fruits and the O.P. developing fruits showed a green and white area at 14 DAT (Figure 1). This was the beginning of the differentiation of two different fruit components, the mesocarp and the endocarp. In the O.P. samples, the endocarp development proceeded through time and the lignification of this tissue was evident at 35 DAT. The same pattern of development was observed in the GA_3_-treated samples.

### 2.2. Initial Development of GA_3_-Treated Samples Follows That Observed in the Open-Pollinated Samples

**Figure 2:**
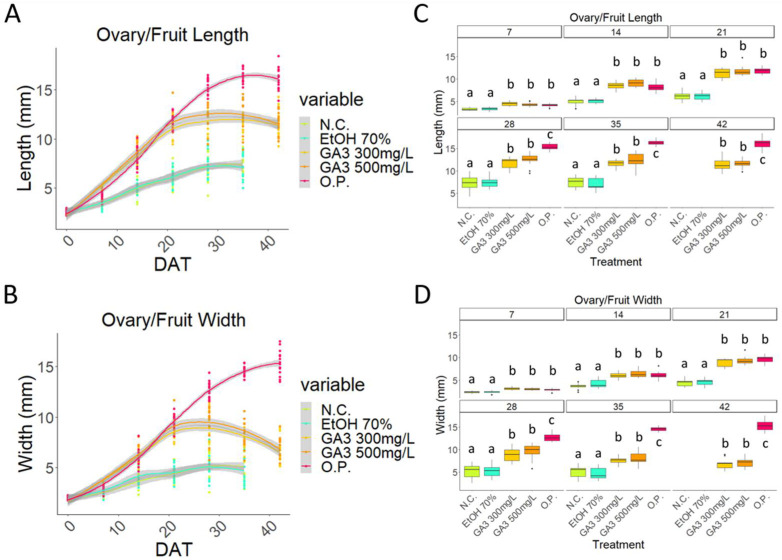
Comparison of unpollinated ovaries (N.C.); EtOH70%-treated ovaries; 300 mg/L GA_3_-treated ovaries; 500 mg/L GA_3_-treated ovaries; and open pollinated (O.P.). Graph that shows the curves obtained from the measurements of (**A**) length and (**B**) width of all the five treatments from the beginning to the end of the experiment; boxplot graph of the (**C**) length measurements and (**D**) width measurements. DAT: days after treatment. Letters indicate significant differences where a is significantly different to b and c and b is significantly different to a and c. Statistical differences indicate lines which are statistically different from each other by *p* < 0.01.

In the 2020 season, measurements of the developing fruits’ length and width showed that the O.P. growth pattern was consistent with a sigmoid curve (Figure 2A,B) [3]. The GA_3_-treated samples showed similar curves, however, only between 0 and 21 DAT, while after this timepoint, a significative difference was visible. Whilst the O.P. 2020 curve continued with its exponential phase, the GA_3_-treated fruit reached a plateau and then a decreasing phase was observed (Figure 2A,B).

Between 7 and 21 DAT, there was no statistically significant difference between the O.P. and GA_3_ samples, while after 28 DAT, a statistically significant was difference observed (Figure 2C,D). The two controls, the negative control and the mock-treated control (EtOH70%), had an almost identical pattern. Very little development occurred, with only a small slope visible, after which, the ovary/fruit were aborted (Figure 2A,B). These two controls always remained statistically smaller compared to the GA_3_-treated and O.P. samples (Figure 2C,D).

**Figure 3:**
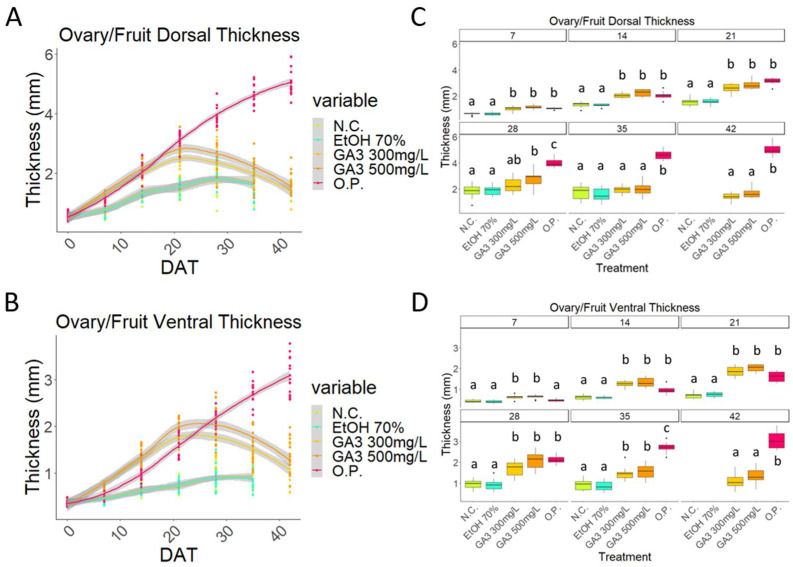
Comparison of unpollinated ovaries (N.C.); EtOH70%-treated ovaries; 300 mg/L GA_3_-treated ovaries; 500 mg/L GA_3_-treated ovaries; and open pollinated (O.P.). Graph that shows the curves obtained from the measurements of (**A**) dorsal wall thickness and (**B**) ventral wall thickness of all five treatments from the beginning to the end of the experiment; boxplot graph of the (**C**) dorsal wall thickness and (**D**) the ventral wall thickness. DAT: days after treatment. Letters indicate significant differences where a is significantly different to b and c and b is significantly different to a and c. Statistical differences indicate lines which are statistically different from each other by *p* < 0.01.

The O.P. dorsal and ventral wall thickness curves appear to be similar to a stretched sigmoid that has not yet reached the plateau phase (Figure 3A,B).

Even when considering these dimensional parameters, a similarity in the pattern of the O.P. and the GA_3_-treated curves can be observed during the early stages of development (0–21 DAT), while the first differences appeared after 21 DAT (Figure 3A,B).

Whilst the walls of the O.P. samples became thicker with time, the GA_3_-treated samples became thicker until timepoint 21 DAT, and then thinner. Interestingly, the GA_3_-treatment curves suggested a more advanced thickness of the ventral wall compared to the O.P. samples (Figure 3). However, a statistically significant difference was present only at time point 7 DAT, while between 14 and 28 DAT, no significant differences were observed (Figure 3C,D). Finally, at 35 DAT, the O.P. samples were significantly thicker than the GA_3_-treated samples (Figure 3C,D).

### 2.3. Mesocarp and Endocarp Development Fits the Pattern of a Double Sigmoid Curve

**Figure 4:**
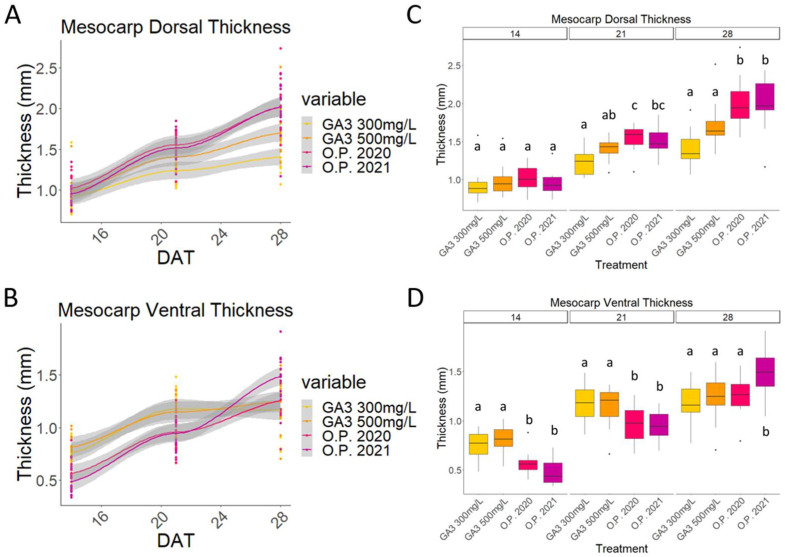
Comparison of ovaries treated with 300 mg/L GA_3_, 500 mg/L GA_3_, and open-pollinated ovaries (O.P.) from 2020 and 2021. Graph that shows the curves obtained from the measurements of (**A**) the mesocarp dorsal thickness and (**B**) the mesocarp ventral thickness of all five treatments from the beginning to the end of the experiment; boxplot graph of the (**C**) mesocarp dorsal thickness and (**D**) mesocarp ventral thickness. DAT: days after treatment. Letters indicate significant differences where a is significantly different to b and c and b is significantly different to a and c. Statistical differences indicate lines which are statistically different from each other by *p* < 0.01.

**Figure 5:**
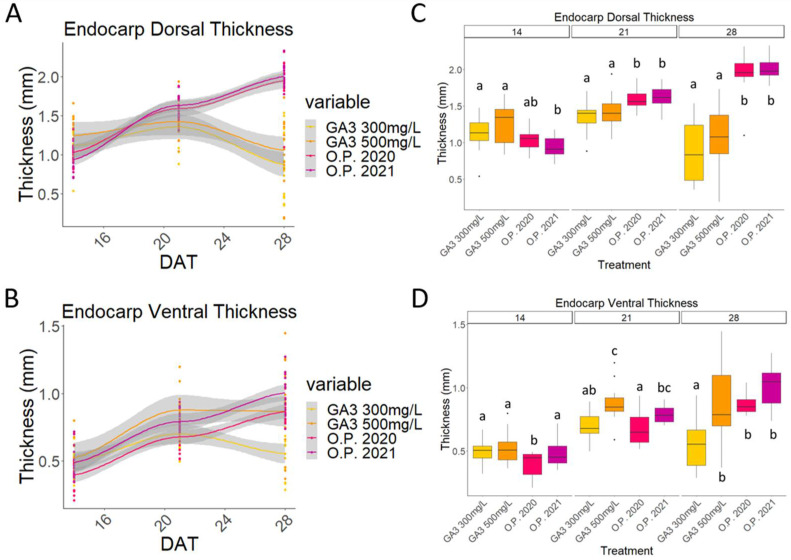
Comparison of ovaries treated with 300 mg/L GA_3_, 500 mg/L GA_3_, and open-pollinated ovaries (O.P.) from 2020 and 2021. Graph that shows the curves obtained from the measurements of (**A**) the endocarp dorsal thickness and (**B**) endocarp ventral thickness of all the five treatments from the beginning to the of end the experiment; boxplot graph of (**C**) the endocarp dorsal thickness and the (**D**) endocarp ventral thickness. DAT: days after treatment. Letters indicate significant differences where a is significantly different to b and c and b is significantly different to a and c. Statistical differences indicate lines which are statistically different from each other by *p* < 0.01.

The patterns of mesocarp and endocarp growth were quite similar if the dorsal and the ventral sides of the fruit are compared. Both followed a clear double sigmoid curve, where three well-marked stages could be identified (Figure 4A,B and Figure 5A,B).

In the first phase (stage I), occurring here between 7 and 19 days, the ovary started to develop. No cell division occurred during phase II (stage II), seen here between 19 and 23 days, where no additional growth could be observed. Finally, in the last phase (stage III), the cells started to enlarge rapidly, contributing to the final fruit size (Figure 4A,B and Figure 5A,B) [3,37].

Differences between the O.P and GA_3_-treated samples can be observed. Firstly, on the dorsal side, the thickness of both the mesocarp and the endocarp were almost the same in the O.P. fruits and in the GA_3_-treated putative fruits (Figure 4A,B and Figure 5A,B). However, the O.P. fruits became thicker than the GA_3_-treated fruit, whose mesocarp grew slightly before wilting and the endocarp degeneration was already visible at 28 DAT (Figure 4 and Figure 5). Secondly, both the ventral mesocarp and the dorsal and ventral endocarp of the GA_3_-treated developing putative fruits were thicker than those of the O.P. developing fruits at 14 DAT (Figure 4C,D and Figure 5C,D). This difference was maintained in the ventral mesocarp until 28 DAT, when no significant difference was observed (Figure 4D), while at 21 DAT, the dorsal mesocarp was significantly thicker in the O.P samples (Figure 4C). Finally, the endocarp showed an interesting characteristic, because, for the first time, a difference between the two dosages of GA_3_ (300 mg/L and 500 mg/L) was observed (Figure 5D). 

Whilst the putative fruits treated with 300 mg/L showed a pattern coherent with that described for the dorsal endocarp (i.e., a decrease in size), significant growth of the ventral endocarp after treatment with 500 mg/L was visible between 14 and 21 DAT. The ventral endocarp was thicker even than that of the O.P. fruit at 14 DAT, while at 21 DAT, the thickness of these two treatments was no longer significantly different (Figure 5C,D).

### 2.4. Impact of Environmental Differences in 2020 and 2021 on the Development of Cherry Fruit cv Regina

**Figure 6:**
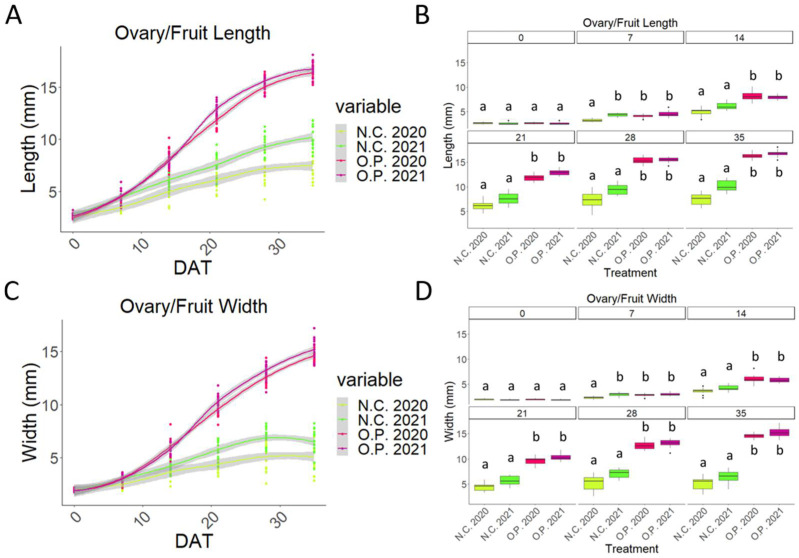
Comparison between ‘Regina’ 2020 and ‘Regina’ 2021 unpollinated ovaries (N.C.) and open pollinated (O.P.) samples over two growing seasons. (**A**) Dorsal wall thickness measurements. (**B**) Boxplot graph of the dorsal wall thickness measurements. (**C**) Ventral wall thickness measurements. (**D**) Boxplot graph of the ventral wall thickness measurements. Days after treatment. Letters indicate significant differences where a is significantly different to b. Statistical differences indicate lines which are statistically different from each other by *p* < 0.01.

**Figure 7:**
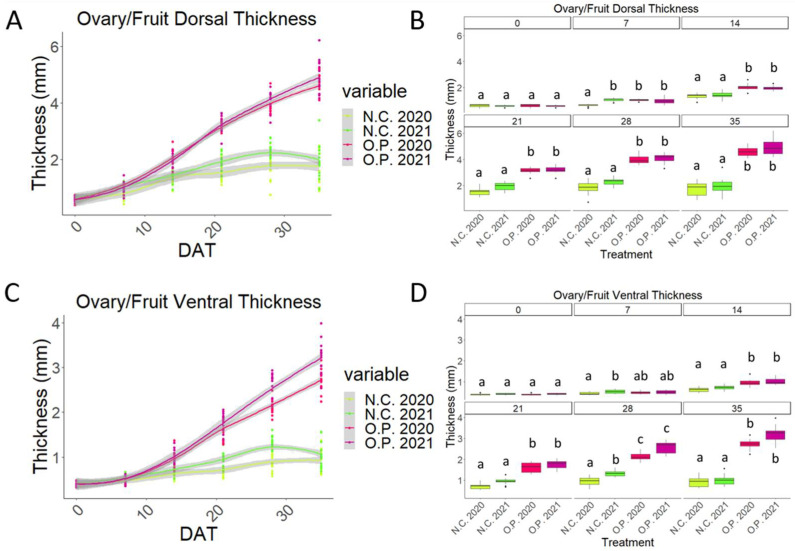
Comparison between ‘Regina’ 2020 and ‘Regina’ 2021 unpollinated ovaries (N.C.) and open pollinated (O.P.) samples over two growing seasons. (**A**) Length. (**B**) Boxplot graph of the length measurements. (**C**) Width measurements. (**D**) Boxplot graph of the width measurements. DAT: days after treatment. Letters indicate significant differences where a is significantly different to b and c and b is significantly different to a and c. Statistical differences indicate lines which are statistically different from each other by *p* < 0.01.

It is important to account for the role that climate plays in cherry tree cultivation and final crop yield. In the case of cherry, this can often result in the loss of a significant proportion of fruits before ripening. This process, referred to as ‘June drop’, varies from year to year, and in some seasons, can result in a total loss of the crop. In the UK in 2000, for example, cherry trees lost as much as 90% of their fruit set before harvest [3,14]. With this in mind, we analysed the growth pattern of unpollinated (N.C.) and open-pollinated (O.P.) sweet cherry (cv Regina) over two growing seasons, 2020 and 2021. The later year, 2021, was characterised by a decrease in rainfall from 104 mm in 2020 to 25 mm in 2021, the maximum temperature was 2 degrees lower in 2021, with an increase from 1 day to 7 days of air frost and a decrease in total sun hours from 75 to 70 h. The patterns of the curves of the N.C. and the O.P. samples from 2020 and 2021 were very similar, although some differences can be observed. Interestingly, while in 2020 at 7 DAT, a significant difference was observed between the N.C. and the O.P. samples, this difference was not observed in 2021 (Figure 6 and Figure 7).

In all the four-dimensional parameters (length, width, and dorsal and ventral thickness), the N.C. 2021 curve was always greater than the N.C. 2020 sample. At some timepoints, this difference was significant. At time point 7, the N.C. 2021 values were always significantly greater than the N.C. 2020 values (Figure 6 and Figure 7). From 14 DAT, a clear separation of the O.P. 2020 and 2021 from the N.C. 2020 and 2021 can be observed. Finally, a small difference can be observed in the N.C 2021 samples compared to the N.C. 2020 ones. Indeed, at 35 DAT and 28 DAT, the N.C. 2021 resulted in being slightly longer and thicker in the ventral wall, respectively, than the N.C. 2020 unpollinated ovaries. This difference was not observed at any timepoint when the O.P. 2020 and O.P. 2021 samples are compared (Figure 6 and Figure 7).

In a comparison of the endocarp thickness of O.P. 2020 vs. 2021, no differences were observed at any timepoint when the O.P. 2020 and O.P. 2021 samples are compared (Figure 6 and Figure 7). The O.P. 2021 fruits displayed a thicker ventral mesocarp compared to the O.P. 2020 fruits, and the difference at some timepoints was statistically significant (Figure 4C,D and Figure 5C,D), whilst the dorsal thickness remained comparable throughout the experiment in these same samples (Figure 4C,D and Figure 5C,D).

## 3. Discussion

### 3.1. GA_3_ Treatment Is Sufficient to Initiate Fruit Set in Sweet Cherry

Gibberellins (GAs) are required for the development of seeds. Previous works have shown that GA-deficient mutants display altered seed development and a significant level of abortions [3,31,33,38,39]. Previous studies on satsuma ovaries have shown that treatment with GA_3_ caused increases in GA_20_ and GA_1_, which are the most important GA precursors [31]. In satsuma and clementine, GA_3_ enhances cell division, increasing the number of cell layers in the pericarp (effect lost after 15 DAT), while treatment with paclobutrazol (PBZ), an inhibitor of GA biosynthesis, has the opposite effect [31]. We showed here that GA_3_-treated cherry ovaries developed into putative fruits, but that their development reached a plateau between 21 and 28 DAT after treatment. By 35 DAT, the fruits began to shrink, indicating that gibberellins are involved in the activation of the cell cycle in ovary wall cells, leading to fruit initiation.

The growth curves obtained from the measurements of the O.P. developing fruits (2020 and 2021) showed a pattern coherent with that reported in the literature. In particular, the length and width of the curves displayed a sigmoid growth pattern typically seen in stone fruit [3,40,41,42,43]. Drupe development followed a double sigmoid pattern, in which three phases were recognised. The observations in ‘Regina’ ended after a month, and the first half of the double sigmoid was obtained in both 2020 and 2021. This is consistent with previous studies, where it was reported that phase I for the mid-season variety ‘Montmorency’ lasts 20–22 days [40]. According to Tukey and Young [37], during phase I, the different tissues are established, and this is what was observed in ‘Regina’ in this study. During phase II, the endocarp starts its lignification process, and again, this is consistent with the observations in this study between 21 and 28 DAT, when the length and width curves reached the plateau. The endocarp started to harden at 28 DAT, which could be the first timepoint included in phase II. At 35 DAT, the lignification of the endocarp was quite visible. Thus, combining the curves obtained and the observations on the endocarp lignification, it is likely that the switch from phase I and phase II occurred between the timepoints of 21 and 28 DAT. Furthermore, timepoint 21 DAT seems crucial, since it was when a difference appeared in the growth curve patterns between the O.P. and GA_3_-treated samples.

### 3.2. The Arrest of the Growth of the GA_3_-Treated Developing Putative Fruits Begins after Twenty-One Days

After 21 DAT, the arrest of the growth of the GA_3_-treated developing putative fruits began. A plateau phase was present also in these curves, however, in this case, this plateau could have a different significance. Indeed, while in the O.P. fruit, the plateau phase was observed because the mitotic divisions were reduced, in favour of cell expansion, while the plateau observed in the GA_3_-treatment curves was likely due to an arrest of the actual development. These data suggest that a separate signal is required at 28 DAT in order for fruit development to successfully continue beyond this point. Despite the inability to complete fruit development, it seems that GA_3_ is involved in fruit set initiation. The putative fruits were observed to be quite similar to the O.P. fruits in the first phases of the experiment before the wilting. It would be helpful to repeat these treatments, with multiple applications over time to check if this approach could lead to mature parthenocarpic cherry fruit. This is a strategy that needs to be considered for future experiments. 

As previously noted, during fruit development, communication between the seed/embryo and the fruit is necessary for development. These signals are believed to involve phytohormones such as auxin and gibberellins produced either in the seed coat, developing embryo, or endosperm [14,44,45,46,47]. This complex inter-hormonal signalling mechanism that identifies an aborting seed to the tree and results in fruit abscission is poorly understood; however, it is probable that either a signal travels from the seed to the tree to induce fruit abscission or there is a loss of signal from the seed coat, embryo, or endosperm that would normally be present during successful seed development. Such a process has been suggested to be involved in ‘June drop’, a process that begins in late June until mid-July and can result in a significant loss of yield. This loss can vary from year to year and is often triggered by adverse environmental conditions due to the presence or absence of a signal from the seed coat, embryo, or endosperm [3,48]. In 2020, in the UK, late frosts resulted in a 90% loss of fruit before harvest [3]. 

The absence of a seed in the GA_3_-treated cherry fruit suggests that the lack of a signal from the seed coat, embryo, or endosperm may have been the trigger for the premature termination of fruit development and fruit abscission during these experiments. Identifying and replacing this signal may be necessary for the formation of a fully formed mature parthenocarpic cherry through hormone treatments. Other options would be to identify the targets of such signals and activate the response through genetic modifications. A recent review has identified potential sources of genetic modification that could result in the formation of parthenocarpic cherry fruits [14], based on results previously reported in tomato [49,50,51,52,53,54,55], apple [56,57], and grape [58,59]. 

### 3.3. Annual Environmentally Induced Variation in Fruit Expansion over Two Growing Seasons

With global increases in temperature of 2 °C, an increase in atmospheric [CO_2_] from 420 ppm to 550 ppm by 2050 [60,61,62,63,64,65], and increases in temperature and rainfall in some areas and reductions in others, it is important to understand how annual environmental conditions will affect cherry fruit development [3,66]. The growth curves obtained from the measurements of the O.P. developing fruits (2020 and 2021) showed a pattern coherent with that reported in the literature. In particular, the length and the width curves displayed a sigmoid pattern. Drupe development followed a double sigmoid pattern, in which three phases were recognised. The observations in ‘Regina’ ended after a month and the first half of the double sigmoid was obtained both in 2020 and 2021. This is consistent with the studies of Tukey and collaborators, where they reported that phase I for the mid-season variety ‘Montmorency’ lasts 20–22 days [40] (see Vignati et al. [3] for review). When the experiments conducted in 2020 and 2021 are compared, a variation in the average values is observed. In particular, the 2021 unpollinated ovaries were bigger than the 2020 ones. Two explanations are possible. Firstly, since the flower buds in 2021 opened significantly later than those in 2020 due to unfavourable conditions (a reduced daily temperature and reduced rainfall), the flower organs had more time to grow. Changes in rainfall may also change soil moisture and soil temperature, impacting development. Water is an important requirement in cherry fruit production and the reduction in rainfall observed in 2021 likely delayed flower opening [67]. Secondly, with the increase in air frost from 1 day to 7 days, cold stress could have promoted a higher growth level of the 2021 ovaries. Biotic and abiotic stress have an impact on plants that may choose specific strategies to face them. Weather conditions, orchard age, and nitrogen fertiliser regimes have previously been shown to influence the yield and quality of sour cherry fruits [68].

## 4. Conclusions

Parthenocarpy (from Greek, “Virgin Fruit”) consists of the development of a fruit without the fertilisation of the ovule, which will develop into the seed. Parthenocarpy is not only commercially interesting, but is an important agricultural trait that can mitigate poor fruit set under conditions unfavourable for pollination [69,70,71]. Seedless fruits are becoming more and more popular and preferred by consumers, and there are also significant financial savings made from the reduced cost of processing; therefore, breeding new parthenocarpic varieties could be highly beneficial. 

Parthenocarpy has a strong connection with phytohormones and, here, we showed that gibberellic acids (GA_3_) act as a trigger for fruit set in sweet cherry and permit the normal formation of fruit up to a period of 28 days. This work also showed that additional signals are required for the continued development of fully mature parthenocarpic fruit in sweet cherry. This contrasts with the results reported in apple, [26,27], grape [28,29], tomato [30], and citrus, where GA_3_ treatment alone was shown to induce parthenocarpic fruit formation. As observed here with sweet cherry, GA_3_ does not induce parthenocarpy in cucumber, where the simultaneous application of GA_4_ and GA_7_ (GA_4+7_) was needed [32]. Furthermore, previous studies have shown that the treatment of “Dangshansuli” pear with GA_4+7_ mimics the effect of pollination, such as up-regulating the expression of auxin efflux carriers and increasing the levels of the auxin Indole-3-acetic acid [14,33]. Furthermore, in “Dangshansuli” pear, emasculated pistils treated with the synthetic auxin 2,4-dichlorophenoxyacetic acid have been shown to produce small parthenocarpic fruits, suggesting that there is a complex interplay between hormones at specific points of fruit development. However, the hormonal interactions required to complete sweet cherry fruit development in the absence of pollination are still not understood. The use of GA_3_ (300 and 500 mg/L) in this work was selected based on previously published works [36,72]. However, we cannot rule out that different concentrations of GA_3_ may elicit similar morphological changes. Future work will look at different hormone regimes, including a lower concentration of GA_3_ applied several times over a longer period of time and the roles of auxin and GA_4+7_ in parthenocarpic fruit formation in sweet cherry.

## 5. Materials and Methods

### 5.1. Plant Material and Growth Conditions

Cherry trees of cv ‘Regina’, which is a late-season variety, were used in the experiments conducted in 2020 and 2021. Regina is a self-sterile variety of German origin. Low productivity with maturation occurring 31 days post-Burlat [3]. Regina produces large sized fruit, good taste and cultivated due to its high resistance to cracking. These varieties are self-incompatible, which means that their ovules cannot be fertilised by pollen produced from the same variety. Cherry flowers were emasculated before they reached the balloon stage and all the flower organs, except for the pistils, were removed. These experiments were performed in a cherry orchard situated in East Malling, Kent, United Kingdom (Latitude: 51°17′26.16″ N; Longitude: 0°26′2.04″ E).

### 5.2. Hormone Solution Preparation and Treatments

The active form of GA used in this study was gibberellic acid 3 (GA_3_), as used in previous studies [36,72]. GA_3_ 300 mg/L and 500 mg/L solutions were prepared, as they have been demonstrated to be effective in inducing parthenocarpic fruit development in Chinese cherry [36]. The GA_3_ (Sigma-Aldrich, St. Louis, MO, USA) solutions were solubilised in 70% ethanol (EtOH 70%). Both solutions (GA_3_ 300 mg/L and GA_3_ 500 mg/L) were prepared and stored at 4 °C. A sprayer containing EtOH 70% was used as mock control. A negative control was added as a further control in the experiments of 2020 and 2021. 

### 5.3. Treatment Procedures 

Unopened flowers at the balloon stage were emasculated and only the pistils were left. All flowers that were at the wrong stage, either too young or already open, were removed from the branch along with the flower buds. The emasculated flowers of each branch were treated in the same way, except for the negative control, which remained untreated: sprayed with the mock solution (EtOH 70%), sprayed with hormone solutions (GA_3_ 300 mg/L and GA_3_ 500 mg/L), or hand-pollinated. The spraying regime consisted of spraying individual flowers a single time at the start of the experiment. No further treatments were carried out. The treated branches were covered with pollination bags in order to prevent access by bees, which are the pollinators of cherry flowers [3,71,73]. In the case of the open pollination, only the flowers at the balloon stage were left on the branch, while all the other stages were discarded. Thus, all the remaining flowers reached the anthesis stage at the same time. Three biological replicates were taken (indicated by the capital letters A, B, and C) Treatments were carried out in triplicate, i.e., one treatment on three different trees. All the treatments were performed on branches that were in close proximity to normalise the treatments as best as possible. 

### 5.4. Sampling and Storage 

The treated pistils were checked every seven days after treatment (DAT). For all treatments, approximately 10 fruits were collected and 5 were used to measure the dimensional features. 

### 5.5. Samples Photo Shooting and Measurements

Ovaries and developing fruits were cut down from the stalk to the tail at the level of the suture line, i.e., the visible line that remained after the closure of the ovary (Figure 1). The fruit were shown to have an uneven localisation of the ovary. It was decided to refer to the thicker ovary/fruit wall where there was the suture line as “dorsal”, and the thinner opposite side as “ventral”. Five ovaries or fruits were photographed per treatment at every timepoint. Measurements of the dimensions were taken using ImageJ1 (https://imagej.net/). 

### 5.6. Statistical Analysis

The data were analysed in R for statistical computing (The R Project for Statistical Computing (https://www.r-project.org/)), where all statistical analyses were carried out, using the Kruskal–Wallis test. Figures were created using R (ggplot2) version 1.52. Statistical differences and *p* = values are given in all figure legends.

## Data Availability

Data supporting reported results can be obtained from the corresponding author.
